# Multi-Faceted Assessment of Liquid Chromatographic Methods for the Analysis of Sulfonamides in Environmental Waters (2020–2026): Towards Greener and More Sustainable Approaches

**DOI:** 10.3390/antibiotics15100981

**Published:** 2026-10-04

**Authors:** Heba Shaaban, Abdulmalik M. Alqarni, Ahmed Mostafa, Turki Aldossary, Noor Bunajmah, Hashim Almusaher, Shmoukh A. Albugami, Ghada Alsumain, Fatimah A. Alansari

**Affiliations:** 1 Department of Pharmaceutical Chemistry, College of Pharmacy, Imam Abdulrahman Bin Faisal University, King Faisal Road, P.O. Box 1982, Dammam 31441, Saudi Arabia; amalqarni@iau.edu.sa (A.M.A.); ammostafa@iau.edu.sa (A.M.); 2 College of Pharmacy, Imam Abdulrahman Bin Faisal University, King Faisal Road, P.O. Box 1982, Dammam 31441, Saudi Arabia; 2220002674@iau.edu.sa (T.A.); 2220004196@iau.edu.sa (N.B.); 2250002461@iau.edu.sa (H.A.); 2220007016@iau.edu.sa (S.A.A.); 2140008293@iau.edu.sa (G.A.); 2220001110@iau.edu.sa (F.A.A.)

**Keywords:** sulfonamides, environmental waters, assessment tools, green analytical chemistry, AGREE, IMOGI

## Abstract

**Background/Objectives**: Sulfonamides are widely used antibiotics that frequently occur as emerging contaminants in environmental waters, raising concerns about their ecological and public health impacts. Although numerous chromatographic methods have been reported for their determination, systematic evaluation of their environmental sustainability, practicality, and innovation remains limited. Therefore, this study aimed to perform a comprehensive multi-criteria assessment of liquid chromatographic methods for sulfonamide determination in environmental waters to support informed method selection and guide the development of greener, more practical, and innovative analytical approaches. **Methods**: Twelve reported liquid chromatographic methods were selected and assessed using five complementary assessment tools: the Analytical GREEnness Metric (AGREE) and Modified Green Analytical Procedure Index (MoGAPI) for environmental sustainability; the Blue Applicability Grade Index (BAGI) and Click Analytical Chemistry Index (CACI) for practicality; and the Innovative Method Orange Gradient Index (IMOGI) for methodological innovation. **Results**: The evaluated methods showed considerable variation across the three assessed dimensions of environmental sustainability, practicality, and methodological innovation. Some methods showed favorable characteristics in specific dimensions, while others incorporated different combinations of environmental sustainability, practicality, and innovation features. **Conclusions**: The use of complementary assessment tools provides a multidimensional view of environmental sustainability, practicality, and methodological innovation without combining scores into a composite measure or formal ranking. The findings highlight opportunities to improve liquid chromatographic methods for sulfonamide determination through more sustainable sample preparation, practical workflows, and methodological innovation.

## 1. Introduction

Sulfonamides are among the most widely used medications due to their ability to treat a variety of human diseases, as well as their extensive application in agriculture, aquaculture, and animal husbandry. Their environmental behavior is influenced by several factors, including physicochemical properties, dosage, and the nature of the environmental components with which they interact [1]. Sulfonamides exhibit considerable structural diversity and a wide range of pharmacological activities, making them a major class of compounds used worldwide. Their antibacterial application represents one of the most prominent uses, as these agents are effective in treating infections caused by pathogenic microorganisms. Notably, sulfonamides ranked as the third most sold class of antimicrobials in European countries in 2018 [2].

Sulfonamides are associated with a range of adverse drug reactions, with hypersensitivity reactions being among the most frequently reported. Numerous clinical reports have highlighted the potential for cross-reactivity with other drugs, which may further impact patient safety [3]. Moreover, patients with AIDS have been reported to experience skin rashes following sulfonamide administration. While many of these reactions are benign, they may progress to severe and potentially life-threatening toxidermias [3]. Additional adverse reactions include acute liver injury, pulmonary complications, and blood dyscrasias [4].

Over the past few decades, there has been a significant increase in pharmaceutical consumption due to the rising prevalence of diseases and the expansion of drug manufacturing. This trend has also been accompanied by population growth and increased demand for animal protein, particularly in agricultural systems [5].Consequently, pharmaceutical residues, including antibiotics such as sulfonamides, have become increasingly prevalent in the environment. Due to their water solubility, sulfonamides can be released into aquatic systems, where they may persist and contribute to potential environmental risks [6]. Antimicrobial resistance is recognized by the World Health Organization as one of the major global public health threats of the 21st century. The extensive use and misuse of antibiotics in human medicine, veterinary practice, and agriculture contribute significantly to the emergence and spread of resistant microorganisms [1]. Sulfonamides, widely used antimicrobial agents, can contribute to this problem by exerting selective pressure on bacterial populations and facilitating the dissemination of resistance genes through horizontal gene transfer. Consequently, they are considered important environmental contaminants associated with the development of multidrug-resistant bacteria [7].

In response to growing environmental concerns, the European Medicines Agency has established strict guidelines requiring the Environmental Risk Assessment (ERA) of medicinal products [8]. This assessment includes a tiered approach involving Phase I and Phase II evaluations to ensure that environmental concentrations of pharmaceuticals remain below the predicted no-effect concentration [8]. Furthermore, several pharmaceutical compounds, including antibiotics, have been included in the EU Water Framework Directive Watch List for Union-wide monitoring [9]. This program aims to improve monitoring data for emerging contaminants in aquatic environments and support the development of strategies for controlling pharmaceutical pollution [10].

Different analytical methods have been developed for the determination of antibiotics, including sulfonamides [11,12,13,14,15]. Most of the reported methods consume large volumes of harmful organic solvents and consequently produce large volumes of waste. Growing awareness of both environmental protection and analysts’ health has increased interest in replacing conventional analytical methods with greener alternatives [16]. These approaches aim to minimize or eliminate the use of organic solvents while maintaining satisfactory chromatographic performance [17].

The adoption of green analytical chemistry (GAC) principles in analytical laboratories has gained considerable attention in recent years. The principles and recommended guidelines of GAC provide a fundamental framework for achieving a balance between efficient analytical performance and environmentally safe procedures. There is a growing effort to replace conventional analytical techniques with more environmentally sustainable alternatives [16]. Various approaches have been proposed to improve the environmental sustainability of pharmaceutical analysis [18,19]. Among the most common strategies are shortening chromatographic run times, employing environmentally benign solvents, and miniaturizing analytical systems [17]. Reducing analysis time can contribute to improving the greenness of pharmaceutical analysis by potentially lowering solvent consumption and waste generation associated with the analytical workflow. However, analysis time should be considered alongside other factors, including sample preparation, solvent and reagent consumption, waste generation, and energy requirements, when evaluating the overall sustainability of an analytical method [20,21]. In addition, replacing hazardous organic solvents in extraction procedures with greener alternatives is an effective strategy to minimize the environmental impact of analytical methods. Substituting traditional organic solvents with more environmentally benign options, such as natural deep eutectic solvents, has recently received increasing attention within the scientific community [22,23,24].

Achieving a balance between analytical efficiency and environmentally safe practices is a key consideration during method development. Various assessment tools have been developed to assess the greenness of analytical methods, such as the Analytical GREEnness Metric (AGREE) [25] and the Modified Green Analytical Procedure Index (MoGAPI) [26]. Beyond environmental assessment, different tools for evaluating the practicality and applicability of the analytical methods were developed, including the Blue Applicability Grade Index (BAGI) [27] and the Click Analytical Chemistry Index (CACI) [28]. Innovative Method Orange Gradient Index (IMOGI) was recently introduced by Shaaban, H., to assess the level of analytical methodological innovation [29]. As each assessment tool, including AGREE, MoGAPI, BAGI, and CACI, evaluates specific parameters and characteristics of chromatographic methods, their combined application provides a broader assessment of the analytical workflow than can be obtained from a single assessment tool. Evaluating the methods from different perspectives also provides insights into the factors that drive their environmental impact, practical applicability, and innovation. Such a multidimensional assessment can therefore reveal the strengths and limitations of different methodological approaches and help identify opportunities for achieving a more balanced analytical method in terms of environmental sustainability, practicality, and innovation [30]. Careful planning and evaluation of the sustainability of analytical procedures during method validation are strongly recommended before routine application to minimize environmental hazards.

Unlike previous studies that have discussed and compared chromatographic methods for the determination of parabens in cosmetic products, primarily focusing on the reported analytical approaches and their characteristics, the present study addresses this gap by providing a multidimensional assessment of these methods from the perspectives of environmental sustainability, practical applicability, and innovation and modernization. To the best of our knowledge, these three dimensions have not previously been systematically evaluated within a common benchmarking framework for paraben determination in cosmetic products. In this study, MoGAPI, BAGI, and IMOGI were applied to provide complementary assessments of environmental sustainability, practical applicability, and innovation and modernization, respectively. Furthermore, the present study goes beyond assigning assessment scores by identifying the methodological factors that contribute to more sustainable and practically applicable analytical workflows, including solvent consumption, choice of extraction media, sample-preparation requirements, and accessibility of materials and reagents. These insights can help guide the future development and improvement of chromatographic methods for paraben determination toward more sustainable, practical, and innovative analytical approaches.

## 2. Results and Discussion

In order to gain a deeper understanding of analytical methods reported for the determination of sulfonamides in environmental waters in terms of their environmental impact, practical applicability, and innovation, several assessment tools were used. The scoring of the 12 selected methods according to AGREE, MoGAPI, CACI, BAGI, and IMOGI was performed based on the criteria and scoring options specified by each metric. The detailed scoring sheets for all criteria and methods are provided in Tables S1–S5.

### 2.1. Assessment of the Environmental Sustainability of the Reported Methods

The environmental sustainability of the liquid chromatographic methods reported for the determination of sulfonamides in environmental water samples was evaluated using two complementary greenness assessment tools, AGREE and MoGAPI. The chromatographic and sample-preparation conditions of the investigated methods, including the extraction procedures, solvents, chromatographic systems, and detection techniques, are summarized in Table 1. Other experimental and instrumental characteristics are provided in Table S6. The use of both metrics provides a broader metric-based evaluation of the environmental profile of the investigated analytical procedures because they consider different aspects of analytical greenness.

The AGREE metric evaluates analytical procedures according to the 12 principles of Green Analytical Chemistry (GAC) and generates a score between 0 and 1, with higher scores indicating better compliance with the GAC principles. The AGREE scores ranged from 0.34 to 0.64, demonstrating differences in the environmental performance of the investigated methods. The detailed AGREE scores and corresponding pictograms are presented in Table S1.

Method 4, reported by Mostafa, A. [34], achieved the highest AGREE score (0.64), indicating the most favorable AGREE profile among the investigated methods. This method employs dispersive liquid–liquid microextraction based on a deep eutectic solvent (DES-DLLME). The miniaturized extraction approach reduces the amount of sample and extraction solvent required, thereby limiting waste generation. In addition, the use of a DES provides an alternative to conventional organic extraction solvents, depending on its composition. These features likely contributed to the favorable environmental profile of Method 4. Although DES-DLLME may offer environmental advantages, these benefits depend on factors such as the composition and amount of DES used and the waste generated during the procedure. Therefore, DES-DLLME should not be considered inherently greener than conventional extraction approaches such as SPE, as its environmental performance ultimately depends on the characteristics of the entire analytical workflow.

In contrast, Method 6 showed the lowest AGREE score (0.34). This method uses solid-phase extraction (SPE), which generally involves conditioning, washing, and elution steps and requires disposable sorbent materials. These additional operations may increase solvent and solid consumption and generate additional waste, contributing to the less favorable AGREE profile observed for Method 6. Most of the remaining methods showed intermediate AGREE scores, mainly between 0.37 and 0.55. This reflects the continued dependence on conventional chromatographic workflows and organic mobile phases, particularly methanol and acetonitrile. Although these solvents may be required to achieve adequate chromatographic performance, their consumption and disposal are among the factors considered in the greenness assessment of LC-based methods.

The MoGAPI assessment provided a complementary evaluation of the analytical procedures, considering aspects related to sample preparation, solvent and reagent consumption, hazards, waste generation, and other methodological characteristics. The MoGAPI scores ranged from 58 to 70 (Table S2). Methods 4 and 5 achieved the highest score (70), whereas Methods 3 and 6 obtained the lowest score (58). The relatively narrow range indicates that the investigated methods share several common characteristics within the dimensions evaluated by MoGAPI. The MoGAPI results further support the favorable profile of Method 4, which combines chromatographic analysis with DES-DLLME sample preparation. Its maximum MoGAPI score, together with the highest AGREE score, indicates consistency between the two metric-based greenness profiles. Conversely, Method 6, based on SPE, received the lowest MoGAPI score, consistent with its low AGREE score. This highlights that sample-preparation characteristics are an important component of the greenness profiles captured by the assessment tools. The two metrics did not produce identical score patterns. For example, Method 5 achieved the same highest MoGAPI score as Method 4 (70) but had a lower AGREE score (0.55 versus 0.64). This difference demonstrates the complementary nature of the two assessment tools and indicates that different aspects and criteria of analytical greenness are emphasized by each metric.

Overall, the investigated methods demonstrated moderate metric-based environmental sustainability profiles, indicating considerable scope for further improvement. The comparison of Methods 4 and 6 illustrates differences in their metric-based greenness profiles: the DES-DLLME-based Method 4 showed the most favorable overall greenness profile, whereas the SPE-based Method 6 showed the least favorable profiles across these metrics. Future method development should therefore emphasize miniaturized extraction, reduced solvent and sorbent consumption, lower waste generation, and more sustainable extraction media, while maintaining the required analytical performance.

### 2.2. Assessment of the Practicality of the Reported Methods

The practical applicability of the investigated methods was evaluated using the CACI and BAGI metrics. The CACI scores ranged from 47 to 70, while BAGI scores ranged from 47.5 to −65, indicating noticeable differences in the operational suitability of the investigated procedures. As shown in Tables S3 and S4, these differences are mainly associated with the scale and complexity of sample preparation, analytical capability, material requirements, and overall workflow efficiency.

Method 8 achieved the highest CACI score (70), indicating the most favorable practicality according to this metric. The method employs micro SPE (µSPE) coupled with LC–MS/MS for the determination of five sulfonamides. Although µSPE still relies on a sorbent cartridge or cartridge-like device, its microscale format substantially reduces the amount of sorbent, sample, and solvent required compared with conventional SPE. This miniaturization can reduce material consumption and simplify the extraction process while retaining the preconcentration benefits of SPE. More importantly, the use of LC–MS/MS provides substantially higher sensitivity than conventional HPLC-PDA detection, allowing trace-level determination with reported LODs of 0.15–0.46 ng/L. However, the use of LC–MS/MS may also involve higher instrument costs, specialized infrastructure, maintenance requirements, and the need for trained personnel; therefore, high sensitivity alone does not necessarily indicate greater practical applicability for routine laboratories. The combination of microscale extraction and highly sensitive detection provides an important analytical advantage, although its practical applicability depends on the overall requirements of the analytical workflow. The comparison of Methods 8 and 9 provides further insight into the factors governing practicality. Both methods use µSPE, but Method 8 employs LC–MS/MS, whereas Method 9 uses HPLC-PDA. The difference in analyte number is relatively small (five versus four) and is therefore unlikely to be a major determinant of their different scores. Instead, the substantially greater sensitivity of LC–MS/MS is a more important distinguishing factor. Method 9 reported LODs of 1000–3000 ng/L., compared with 0.15–0.46 ng/L for Method 8. Thus, Method 8 provides considerably greater sensitivity while retaining the advantages of microscale sample preparation. This difference is reflected in its higher CACI score (70 versus 60) and BAGI score (60 versus 55), demonstrating that instrumental capability can substantially influence the practical value of a miniaturized sample-preparation approach. The reported LOD values should be interpreted with caution because direct comparison of sensitivity across the reviewed methods is limited by differences in target analytes, sample matrices, analytical platforms, sample-preparation procedures, enrichment factors, and LOD calculation approaches. Therefore, LOD values were considered only as an indicator of the sensitivity criterion within the CACI assessment and were not used to establish a direct sensitivity ranking among the methods.

Methods 2, 4, and 5 achieved the highest BAGI score (65, 62.5, and 62.5, respectively), with all three methods employing DLLME. The practical advantage of DLLME arises primarily from its inherently small extraction scale, which requires relatively small amounts of sample and extraction solvent and avoids the multistep conditioning, washing, and elution sequence typically associated with conventional SPE. Consequently, DLLME can provide a relatively simple workflow with lower material and operational requirements. This is particularly advantageous for routine analysis, where minimizing preparation time and consumable use can improve laboratory efficiency.

Method 10 showed the lowest CACI score (47) and one of the lowest BAGI scores (47.5). Its poor practicality is mainly driven by the use of materials that are not commonly available in all laboratories, together with a multistep sample-preparation procedure. These characteristics increase the resources and analyst effort required to perform the analysis and can make routine implementation more difficult. The combination of material accessibility and procedural complexity therefore appears to be the main limitation affecting the applicability of Method 10.

The BAGI results also identified Methods 3and 10 as the lowest-scoring methods (47.5). The consistency of the low BAGI and CACI performance suggests that operational complexity and resource requirements can negatively affect applicability. However, the comparison of Method 8 with Methods 3 and 10 also demonstrates that workflow simplicity and instrumental capability contribute to practicality. Differences in the number of preparation steps, handling requirements, and instrumental configuration can influence the overall practicality of the analytical workflow. This highlights that practicality is not determined solely by the extraction technique itself but also by how efficiently the sample-preparation procedure integrates with the analytical platform and can be implemented in routine analysis.

Overall, the practicality assessment indicates that the principal drivers of high applicability are sample-preparation miniaturization, streamlined workflows, efficient use of materials, and sensitive analytical detection. Method 8 demonstrates that µSPE can provide a practical alternative to conventional SPE when its reduced scale is combined with highly sensitive LC–MS/MS detection. Conversely, extensive preparation steps and less accessible materials, particularly in Method 10, can reduce routine applicability. These findings support the development of methods that combine miniaturized sample preparation with sensitive detection and simplified operational procedures to improve their suitability for routine environmental analysis.

### 2.3. Assessment of the Innovation and Modernization of the Reported Methods

The methodological innovation and modernization of the investigated analytical procedures were evaluated using the Innovative Method Orange Gradient Index (IMOGI). IMOGI considers multiple dimensions of analytical innovation, including automation, miniaturization, advanced technologies, AI integration, adaptability and scalability, novel concepts or mechanisms, performance improvement, interdisciplinary capabilities, and compliance with GAC principles. Based on the IMOGI classification, methods scoring > 50 are considered highly innovative, those scoring 30–50 are acceptably innovative, and those scoring < 30 are considered low in innovation. The investigated methods obtained scores ranging from 25 to 55 (Table S5), demonstrating different levels of methodological innovation.

Methods 4 and 10 achieved the highest IMOGI score of 55, placing both in the highly innovative category. Method 4 incorporates several innovation dimensions, including the use of a natural deep eutectic solvent (NADES) as a benign alternative extraction medium, combined with miniaturized DLLME. In addition, GAC principles were considered and evaluated using recognized assessment tools, providing an evidence-based assessment of the sustainability of the developed procedure. The combination of a novel extraction medium, miniaturization, and formal evaluation of analytical sustainability therefore reflects the integration of multiple innovation dimensions considered by IMOGI. Its IMOGI profile reflects the use of solid-phase microextraction (SPME) as a modern miniaturized sample-preparation technique together with LC–MS/MS instrumentation. The integration of modern sample preparation with LC–MS/MS analysis represents methodological modernization within the dimensions considered by IMOGI. However, its high innovation score does not necessarily indicate high practicality, demonstrating that innovation and operational applicability are distinct characteristics of analytical methods.

Method 8 obtained an IMOGI score of 50, placing it at the upper boundary of the acceptably innovative category. The method combines µSPE with LC–MS/MS, integrating sample-preparation miniaturization with LC–MS/MS analysis. Although these features represent clear modernization, its score remained at the acceptance threshold rather than exceeding the criterion for highly innovative methods, indicating that fewer IMOGI innovation dimensions were incorporated compared with Methods 4 and 10. The remaining methods with scores between 30 and 45 were classified as acceptably innovative. Their scores indicate the presence of some modern or innovative characteristics, but without the same degree of integration of the innovation dimensions considered by IMOGI observed in Methods 4 and 10. In particular, methods relying on conventional SPE or classical LC-UV/PDA primarily employ established analytical technologies, with fewer of the modernization features captured by IMOGI than approaches incorporating miniaturized extraction or advanced LC–MS/MS analysis.

In contrast, Methods 9 and 12 obtained scores of 25, placing them in the low innovation category. Method 9 employs µSPE, providing a degree of sample preparation miniaturization; however, its use of HPLC-UV/PDA, a relatively established detection platform, provides fewer modernization features within the IMOGI framework. The IMOGI score therefore reflects the limited integration of additional innovation dimensions beyond sample-preparation miniaturization. Similarly, Method 12 incorporated limited modern methodological features, while its analytical performance remained comparable to existing methods without a clear improvement in analytical capability. Thus, the use of a single modern feature does not necessarily constitute substantial innovation when it is not accompanied by broader methodological advancement or improved analytical capability.

Based on the IMOGI findings, a clear future direction emerges toward strengthening methodological innovation and improving alignment with green analytical chemistry (GAC) principles. Although most methods were acceptably innovative, and only a few achieved high innovation scores, the overall distribution indicates that innovation in the field is predominantly incremental within the dimensions evaluated by IMOGI rather than reflecting broad integration of multiple innovation dimensions.

### 2.4. Integrated Assessment and Comparative Evaluation of the Reported Methods

**Figure 1:**
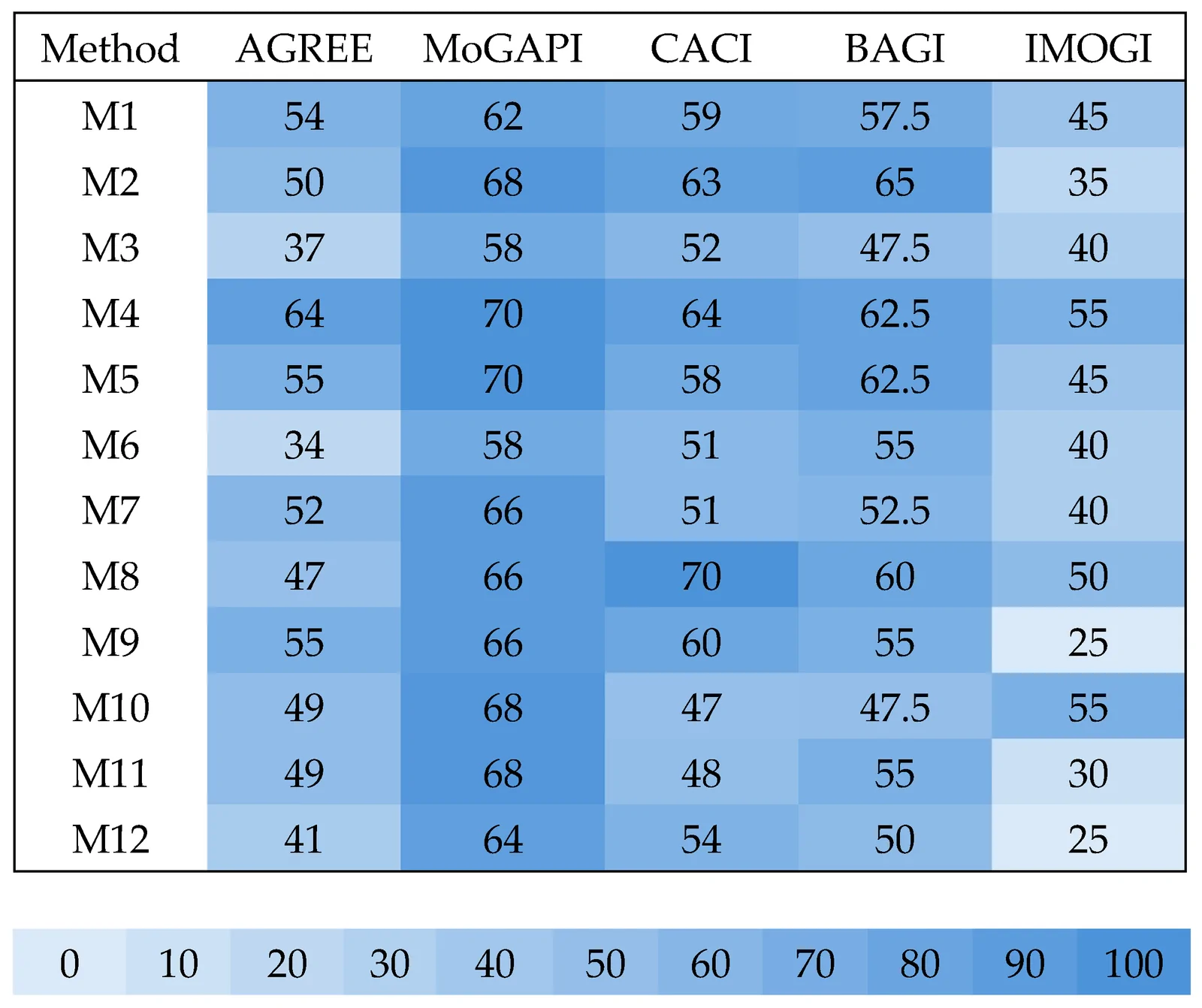
Heat map visualization of the individual scores obtained for the 12 reported liquid chromatographic methods using AGREE, MoGAPI, CACI, BAGI, and IMOGI. AGREE scores were converted from the original 0–1 scale to a 0–100 scale for visualization purposes. The individual assessment scores were not mathematically combined or aggregated.

The integrated assessment provides an overall perspective on the environmental sustainability, practical applicability, and methodological innovation of the investigated methods. The results demonstrate that these dimensions do not necessarily improve in parallel, and therefore no single assessment metric can fully describe the overall quality of an analytical procedure. As illustrated in Figure 1, the differences in the individual scores reflect distinct methodological strengths and limitations across the investigated workflows.

Importantly, each assessment tool evaluates the analytical method according to predefined criteria and from a specific perspective. Thus, a particular methodological feature may contribute differently to the individual assessment dimensions. For example, the incorporation of an advanced separation technique or innovative sample-preparation approach may contribute positively to the IMOGI score, while additional processing steps or operational requirements associated with the reported procedure may result in a lower BAGI score. Such differences do not represent contradictory findings; rather, they provide complementary information on different aspects of the same analytical workflow. This multidimensional approach therefore provides a broader basis for identifying opportunities to achieve more balanced analytical practices by considering environmental sustainability, practical applicability, and innovation together.

A particularly important finding is that Methods 4, 8, and 10 represent different approaches to methodological advancement. Method 4 combines sustainability-oriented extraction chemistry with miniaturization, Method 8 combines microscale extraction with highly sensitive instrumental analysis, while Method 10 emphasizes modern sample preparation and advanced detection. These approaches demonstrate that analytical development can follow different pathways depending on whether the primary objective is to reduce environmental burden, enhance analytical capability, or introduce technological advancement.

The integrated results also highlight an important trade-off between the three assessment dimensions. Advanced instrumentation can provide superior analytical capability but may introduce greater resource or energy requirements, whereas simpler workflows may offer better operational accessibility without providing comparable sensitivity or technological advancement. Similarly, a method incorporating an innovative concept may still require improvement in practical implementation. Therefore, the most desirable methods are not necessarily those achieving the highest score in a single dimension, but those providing a balanced combination of analytical capability, sustainability, practicality, and innovation.

It should also be recognized that the integrated assessment combines results from metrics with different criteria, scoring systems, and assessment boundaries. The scores are therefore presented as complementary profiles rather than as a composite measure or direct ranking of the investigated methods. In particular, AGREE and MoGAPI capture defined aspects of analytical greenness and do not constitute complete life-cycle environmental assessments; factors outside their respective boundaries may not be fully represented.

Overall, the comparative evaluation demonstrates the value of considering these dimensions together. The use of complementary assessment tools provides a more informative characterization of the investigated methods and helps identify the specific methodological features that contribute to their strengths and limitations. This integrated perspective can therefore support more rational selection of analytical procedures according to the requirements of environmental monitoring and guide the design of improved methods.

### 2.5. Future Perspectives

The findings of this study indicate several priorities for the future development of sulfonamide determination methods in environmental waters. Future analytical strategies should increasingly adopt a design-for-sustainability approach, in which environmental impact, practical implementation, innovation, and analytical performance are considered during method development rather than assessed only after the method has been established.

One important direction is the further development of miniaturized extraction approaches using safer and more sustainable extraction media. Reducing the scale of sample preparation can decrease sample, solvent, and sorbent requirements, while alternative extraction media such as NADES can provide opportunities to replace less desirable conventional solvents. However, their benefits should be demonstrated quantitatively using recognized assessment approaches.

Future methods should also seek better integration of advanced analytical technologies with efficient sample preparation. Highly sensitive techniques such as LC–MS/MS remain important for trace-level environmental analysis, but their use should be accompanied by efforts to reduce unnecessary resource consumption and simplify the preceding sample-preparation workflow. The objective should therefore be technological advancement without introducing disproportionate operational or environmental burdens.

Automation and digitalization represent additional opportunities for improving future workflows. Automated or online sample preparation could reduce manual intervention and improve reproducibility and throughput, while digital tools and AI-supported data processing could facilitate data interpretation, method optimization, and high-throughput analysis. These technologies should, however, be incorporated where they provide demonstrable improvements in analytical efficiency or reliability. Greater attention should also be given to method transferability and scalability. Future studies should report practical factors such as sample throughput, material accessibility, cost, operator requirements, and compatibility with routine laboratory infrastructure. This would help distinguish methods that are technically innovative at the research scale from those that can realistically be adopted for routine environmental monitoring.

Finally, future method development should move toward multicriteria optimization, where analytical performance is optimized simultaneously with environmental, practical, and innovation-related characteristics. The combined use of complementary assessment tools can help identify trade-offs during method development and prevent improvements in one dimension from being achieved at the expense of another. Such an approach would facilitate the development of next-generation analytical methods that are sensitive and reliable while also being greener, practically feasible, and technologically progressive.

## 3. Materials and Methods

### 3.1. Literature Search Strategy and Method Selection

**Figure 2:**
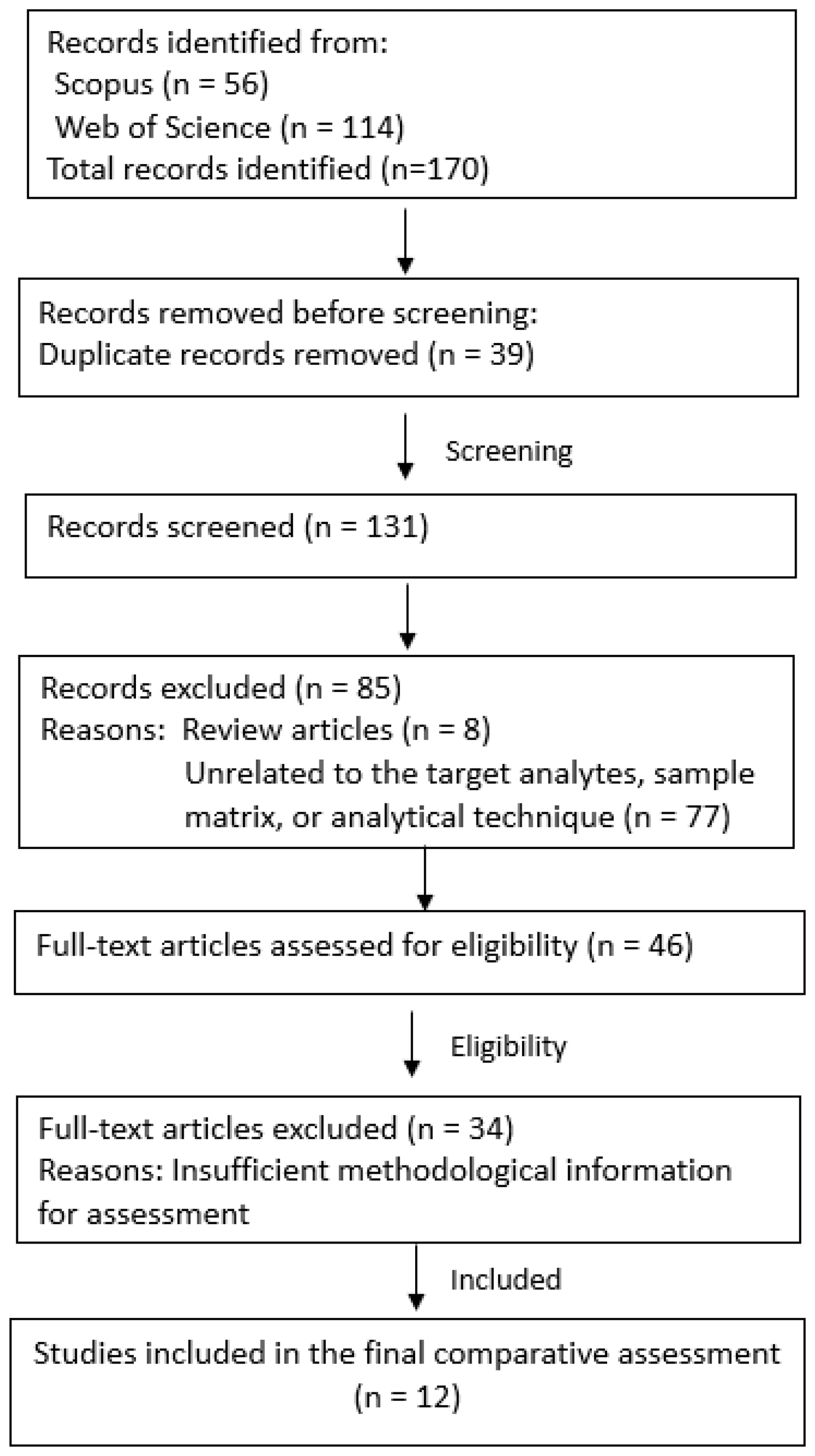
PRISMA flow diagram of the literature search and study selection process for liquid chromatographic methods for sulfonamide determination.

A literature search was conducted in Scopus and Web of Science to identify recent liquid chromatographic methods for the determination of sulfonamides in environmental water samples. The search was restricted to studies published from 2020 to August 2026 to focus the assessment on recent developments in sample preparation, chromatographic separation, detection, and more sustainable analytical approaches. The temporal filter was applied strictly to the target matrix of the evaluated methods. This period was selected to provide a contemporary basis for comparing the greenness, practicality, analytical performance, and methodological innovation of currently relevant methods. The literature identification and screening process followed the PRISMA framework, as presented in Figure 2.

Studies were considered eligible if they met all of the following criteria: (i) investigated sulfonamides in environmental water matrices relevant to environmental monitoring, (ii) employed a liquid chromatography-based separation technique, including HPLC, UHPLC, or LC–MS/MS; (iii) were original research articles; and (iv) provided sufficient methodological information to permit objective assessment using the selected analytical assessment tools. In particular, adequate information on sample preparation, solvent and reagent use, chromatographic conditions, detection, and analytical performance was required. Reviews and other non-original publications, studies involving non-target analytes or matrices, methods not based on liquid chromatography, and publications lacking sufficient methodological information for assessment were excluded. Three authors conducted the literature search and screened the identified records against the predefined eligibility criteria. Potentially eligible studies were subsequently assessed by full-text review. Any uncertainties or disagreements regarding eligibility or methodological information were discussed among the authors and resolved by consensus. When a specific parameter required for assessment was not explicitly reported, but could be reliably obtained or derived from the published information, the parameter was estimated accordingly. For example, when chromatographic analysis time was not explicitly stated, it was estimated from the chromatogram using the reported time scale. Following application of these criteria, 12 liquid chromatographic methods were selected for the final comparative assessment. Relevant methodological information was extracted from the original publications, including sample matrix, sample preparation and extraction procedures, solvents and reagents, chromatographic conditions, detection system, analysis time, and reported analytical performance. These parameters were subsequently used for the multi-criteria evaluation of the selected methods using established assessment tools. To minimize potential assessment bias, the 12 analytical methods were independently scored by three co-authors who were not involved in the development of Method 4 or the IMOGI metric and had no related non-financial academic interests. Any discrepancies in scoring were discussed among the assessors and resolved by consensus.

### 3.2. Assessment Tools

The selected liquid chromatographic methods were evaluated using five complementary assessment tools covering three dimensions: environmental sustainability, applicability and practicality, and methodological innovation.

Environmental sustainability was assessed using the Analytical GREEnness Metric (AGREE) [25] and the Modified Green Analytical Procedure Index (MoGAPI) [26].

Practical applicability was evaluated using the Blue Applicability Grade Index (BAGI) [27] and the Click Analytical Chemistry Index (CACI) [28], while the Innovative Method Orange Gradient Index (IMOGI) [29] was used to assess methodological innovation.

#### 3.2.1. Evaluation of Environmental Sustainability

The environmental sustainability of the selected methods was assessed using AGREE [25] and MoGAPI [26]. These tools evaluate different aspects of an analytical procedure that contribute to its environmental impact, allowing systematic comparison of the relative greenness of the selected methods.

AGREE evaluates analytical procedures according to the 12 principles of GAC [25]. The assessment is performed using the freely accessible AGREE software, which generates a circular pictogram divided into 12 segments corresponding to the GAC principles. Each segment is displayed on a red-to-green scale according to the degree of compliance with the respective principle. An overall score ranging from 0 to 1 is generated, with higher values indicating better overall greenness.

MoGAPI provides a detailed evaluation of the environmental aspects of the analytical workflow [26]. The tool considers different stages and components of the procedure, including sample collection and preparation, reagents and solvents, instrumentation, and waste generation. The assessed parameters are represented using a color-coded pictogram and associated scores, ranging from lower to higher environmental impact. The resulting score and pictogram were used to compare the environmental performance of the selected methods.

#### 3.2.2. Evaluation of Applicability and Practicality

The applicability and practicality of the selected analytical methods were assessed using the Blue Applicability Grade Index (BAGI) [27] and the Click Analytical Chemistry Index (CACI) [28]. These tools provide complementary evaluations of the operational characteristics and practical suitability of analytical procedures.

BAGI evaluates the applicability of an analytical method using ten predefined criteria, including the type of analysis, single- or multi-element analysis, analytical technique, simultaneous sample preparation, sample-preparation requirements, sample throughput, reagents and materials, preconcentration, degree of automation, and sample amount [27]. The resulting score, expressed on a 0–100 scale, was used to compare the practical applicability of the selected methods, with higher scores indicating greater applicability.

CACI was used as a complementary tool to evaluate the practical and operational characteristics of the analytical procedures [28]. The CACI assessment considers sample size, level and time of sample preparation, availability of chemicals and reagents, instrument availability, cost per sample, application type, number of analytes and matrices, portability, degree of automation, sensitivity, and sample analysis time. The CACI results were used together with BAGI to provide a broader evaluation of the practical applicability of the selected methods.

#### 3.2.3. Evaluation of Methodological Innovation

The Innovative Method Orange Gradient Index (IMOGI) was used to assess the innovation and modernization level of the selected analytical methods [29]. IMOGI evaluates analytical procedures using 10 predefined criteria addressing aspects of methodological advancement, technological integration, automation, miniaturization, and other features associated with modern analytical practice. Each criterion was scored according to the predefined IMOGI assessment scheme, and the resulting scores were combined to obtain an overall score ranging from 0 to 100. Higher scores indicate a greater degree of methodological innovation and modernization. The results are presented using the IMOGI orange-gradient graphical format, facilitating comparison among the selected methods.

### 3.3. Application of the Assessment Tools

The analytical characteristics of the selected liquid chromatographic methods for the determination of sulfonamides in environmental waters were compiled, including sample matrix, sample preparation procedure, chromatographic system and conditions, detection technique, and analysis time. The five assessment tools—AGREE, MoGAPI, BAGI, CACI, and IMOGI—were subsequently applied individually to each selected method according to their respective assessment criteria and scoring procedures. The resulting scores were used for comparative evaluation of environmental sustainability, practical applicability, and methodological innovation. The assessment results and graphical outputs are presented in Table 2.

## 4. Conclusions

This study presents a multi-metric framework for the comparative evaluation of liquid chromatographic methods for sulfonamide determination in environmental waters. The findings demonstrate considerable variation among methods depending on the assessment perspective, highlighting that environmental sustainability, practical applicability, and methodological innovation are distinct but complementary dimensions of analytical performance. The assessment revealed important methodological features affecting these dimensions, including solvent selection, sample preparation requirements, energy consumption, operational complexity, and the degree of methodological modernization. The results further demonstrate that no single metric can comprehensively characterize these distinct aspects of an analytical method. Accordingly, the complementary assessment profiles can provide evidence to support method selection and guide the development and improvement of analytical procedures addressing specific requirements for greenness, practicality, and innovation.

## Figures and Tables

**Table 1:** Chromatographic conditions for liquid chromatographic methods reported for the analysis of sulfonamides in environmental waters.

No.	Matrix	No. of Analytes	Separation Technique	Extraction	Solvents and Reagents	LOD	Ref.
1	Tap, river and sea water	17 sulfonamides	LC–MS/MS	on-line SPE	Acetonitrile, ammonium acetate, formic acid, methanol, acetone	0.01–0.05 ng/L	[31]
2	Seawater, wastewater, lake water, seafood samples (pomfrets and prawns)	7 sulfonamides	HPLC-DAD	DLLME	Acetonitrile, tetrachloroethane, formic acid	700–7800 ng/L	[32]
3	River water, food samples (egg and cake)	11 sulfonamides	UPLC-MS/MS	Magnetic SPE	Acetonitrile, formic acid, ethylene glycol, FeCl_3_.6H_2_O, CH_3_- COONa.3H_2_O, ethanol, ammonia, 1,4-dioxane, 2,6-pyridine dicarboxaldehyde, 1,3,5-tris(4-aminobenzene)benzene, NaCl, acetic acid	1–78 ng/L	[33]
4	Mineral water, sea water, tap water	4 sulfonamides	UHPLC-DAD	DES-DLLME	Acetonitrile, ethanol, thymol, formic acid, acetic acid, NaCl	780–3420 ng/L	[34]
5	River water	6 sulfonamides	HPLC-PDA	DES-DLLME	Methanol, formic acid, acetonitrile, benzyltriethylammonium chloride, thymol	300–1100 ng/L	[35]
6	Surface water (lakes, streams, brooks)	8 sulfonamides	UHPLC-MS/MS	SPE	Methanol, ammonia, formic acid, acetonitrile, acetic acid, hydrochloric acid	0.03-0.27 ng/L	[36]
7	Tap water, river water	4 sulfonamides	LC-UV	Magnetic (nanoparticles) SPE	Methanol, acetonitrile, sodium dihydrogen phosphate monohydrate, ammonia, Iron (III) chloride hexahydrate, iron (II) sulfate heptahydrate, sodium chloride, sodium hydroxide	1400–3800 ng/L	[37]
8	Tap water, lake water	5 sulfonamides	HPLC-MS/MS	μ-SPE	Methanol, formic acid, Na_2_CO_3_, polyacrylonitrile, benzoin dimethyl ether, 1-ally-3-methylimidazolium chloride, cysteamine hydrochloride, thiabendazole, benomyl, humic acid	0.15–0.46 ng/L	[38]
9	Surface water and honey	4 sulfonamides	HPLC-PDA	µ-SPE	Methanol, acetonitrile, Cetyltrimethylammonium bromide	1000–3000 ng/L	[39]
10	Wastewater, river water, tap water	10 sulfonamides	HPLC-MS/MS	SPME	Methanol, acetonitrile, NaCl, 4-vinylbenzoic acid, 4-vinylphenylboronic acid	0.31-2.3 ng/L	[40]
11	lake water, egg	12 sulfonamides	HPLC-DAD	μ-SPE	Acetonitrile, formic acid, zinc nitrate, iron (III) chloride, sodium hydroxide, ethanol, sodium sulfate, zinc ferrites	LOQ * 60–110ng/L	[41]
12	Wastewater, tap water	7 sulfonamides	HPLC-DAD	3D-printed device coated with SPE resin	Acetonitrile, methanol, formic acid, ammonia, polyvinylidene fluoride, dimethylformamide, hydrochloric acid, sodium hydroxide	600–6000 ng/L	[42]

* LOD was not reported in the original study; the reported LOQ is provided as an informative indicator of sensitivity. SPE: Solid phase extraction. μ-SPE: Micro solid phase extraction. SPME: Solid phase microextraction. DES-DLLME: Deep eutectic solvent-dispersive liquid–liquid microextraction. LC–MS/MS: Liquid chromatography–tandem mass spectrometry. HPLC-DAD: High-performance liquid chromatography-diode array detection. UPLC: Ultra-performance liquid chromatography. UHPLC: Ultra-high-performance liquid chromatography.

**Table 2:** Scores of the reported liquid chromatographic methods for the analysis of sulfonamides in environmental waters using AGREE, MoGAPI, CACI, BAGI, and IMOGI tools.

NO.	AGREE	MoGAPI	CACI	BAGI	IMOGI	Ref.
1	0.54	62	59	57.5	45	[31]
2	0.5	68	63	65	35	[32]
3	0.37	58	52	47.5	40	[33]
4	0.64	70	64	62.5	55	[34]
5	0.55	70	58	62.5	45	[35]
6	0.34	58	51	55	40	[36]
7	0.52	66	51	52.5	40	[37]
8	0.47	66	70	60	50	[38]
9	0.55	66	60	55	25	[39]
10	0.49	68	47	47.5	55	[40]
11	0.49	68	48	55	30	[41]
12	0.41	64	54	50	25	[42]

## Data Availability

The data presented in this study are contained within the article.

## Supplementary Materials

The following supporting information can be downloaded at: https://www.mdpi.com/article/10.3390/antibiotics15100981/s1, Table S1. Detailed criterion-level assessment of the selected methods using AGREE; Table S2. Detailed criterion-level assessment of the selected methods using MoGAPI; Table S3. Detailed criterion-level assessment of the selected methods using CACI; Table S4. Detailed criterion-level assessment of the selected methods using BAGI; Table S5. Detailed criterion-level assessment of the selected methods using IMOGI; Table S6. Experimental and instrumental characteristics of the selected analytical methods for paraben determination. Table S7. Database-specific literature search strategies and study identification.
