# Supplementary material for: Multi-Faceted Assessment of Liquid Chromatographic Methods for the Analysis of Sulfonamides in Environmental Waters (2020–2026): Towards Greener and More Sustainable Approaches
Source: Antibiotics (Basel). 2026 Oct 4;15(10):981. doi: 10.3390/antibiotics15100981 (PMC13700588; doi:10.3390/antibiotics15100981)
Supplement: Supplementary file 1 [file antibiotics-15-00981-s001.zip › antibiotics-4550404-supplementary.pdf]

Supplementary Material

Multi-Faceted Assessment of liquid chromatographic methods for the analysis of sulfonamides in Environmental Waters: Towards greener and more sustainable approaches

Table S1. Detailed criterion-level assessment of the selected methods using AGREE.

#	The Sampling procedures	The Amount of sample	The positioning of the analytical device	No. of steps in sample preparation procedures	Degree of automation	Derivatization agent (if used)	The amount of waste	Number of analytes; sample throughput	The most energy-intensive technique	The type of reagents	Involving the use of toxic solvents or reagents	The threats which are not avoided	Score 0-1 Pictogram
M1	Offline analysis	1 L	Online (online SPE)	4 steps (filtration, extraction, evaporation, reconstitution)	Automatic (online SPE)	None	9 mL	No. of analytes: 7; throughput: 8.6	LC-MS/MS	None of the reagents from biobased sources	Toxic solvents involved	Toxic to the aquatic environment, bioaccumulative, persistent	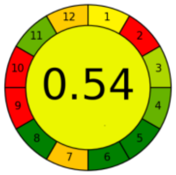
M2	Offline analysis	5 mL	Offline	5 steps (ultrasonication, Centrifugation, drying, reconstitution, filtration)	Semi-automatic (HPLC autosampler)	None	1.7 mL	No. of analytes: 7; throughput: 3.75	HPLC-UV	None of the reagents from biobased sources	Toxic solvents involved	Toxic to the aquatic environment, bioaccumulative, persistent	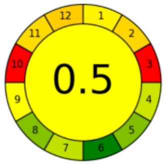
M3	Offline analysis	10 mL	Offline	5 steps (preparation of covalent organic framework, extraction MSPE, drying, reconstitution, filtration)	Semi-automatic (HPLC autosampler)	None	17 mL	No. of analytes: 11; throughput: 5.5	LC-MS/MS	None of the reagents from biobased sources	Toxic solvents involved	Toxic to the aquatic environment, bioaccumulative, persistent	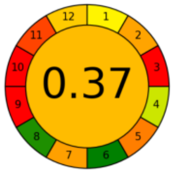
M4	Offline analysis	5 mL	Offline	3 steps (Centrifugation, vortex mixing, filtration)	Semi-automatic (HPLC autosampler)	None	1.65 mL	No. of analytes: 4; throughput: 30	HPLC-UV	Some solvents are bio-based (thymol)	Toxic solvents involved	Toxic to the aquatic environment, bioaccumulative, persistent	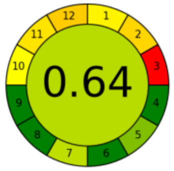
M5	Offline analysis	11 mL	Offline	4 steps (Centrifugation, vortex mixing, dilution, filtration	Semi-automatic (HPLC autosampler)	None	9 mL	No. of analytes: 6; throughput:4	HPLC-UV	Some solvents are bio-based (thymol)	Toxic solvents involved	Toxic to the aquatic environment, bioaccumulative, persistent	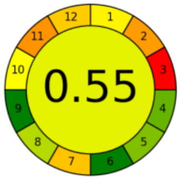

M6	Offline analysis	1 L	Offline	4 steps (extraction, evaporation, filtration, reconstitution)	Semi-automatic (HPLC autosampler)	None	32 mL	No. of analytes:8; throughput:3	LC-MS/MS	None of the reagents from biobased sources	Toxic solvents involved	Toxic to the aquatic environment, bioaccumulative, persistent	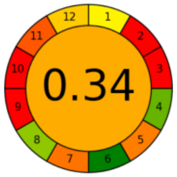
M7	Offline analysis	5 mL	Offline	5 (Synthesis of nanocomposite: stirring, heating, washing), MSPE (sonication, back extraction)	Semi-automatic (HPLC autosampler)	None	1.1 mL	No. of analytes:4; throughput:12	HPLC-UV	None of the reagents from biobased sources	Toxic solvents involved	Toxic to the aquatic environment, bioaccumulative, persistent	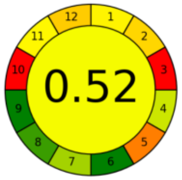
M8	Offline analysis	10 mL	Offline	4 steps (preparation of homemade cartridges)	Semi-automatic (HPLC autosampler)	None	1 mL	No. of analytes:5; throughput:4	LC-MS/MS	None of the reagents from biobased sources	Toxic solvents involved	Toxic to the aquatic environment, bioaccumulative, persistent	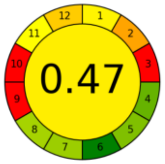
M9	Offline analysis	10 mL	Offline	4 steps	Semi-automatic (HPLC autosampler)	None	0.65 mL	No. of analytes:4; throughput:4	LC-UV	None of the reagents from biobased sources	Toxic solvents involved	Toxic to the aquatic environment, bioaccumulative, persistent	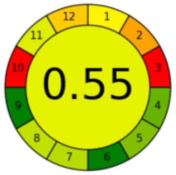
M10	Offline analysis	20 mL	Offline	4 steps (two steps of fabrication of PMA/ MMF-SPME, evaporation, reconstitution)	Semi-automatic (HPLC autosampler)	None	0.5 mL	No. of analytes:10; throughput:5	LC-MS/MS	None of the reagents from biobased sources	Toxic solvents involved	Toxic to the aquatic environment, bioaccumulative, persistent	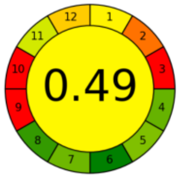
M11	Offline analysis	20 mL	Offline	4 steps (extraction, sonication, evaporation, dilution)	Semi-automatic (HPLC autosampler)	None	2 mL	No. of analytes:12; throughput:2	LC-UV	None of the reagents from biobased sources	Toxic solvents involved	Toxic to the aquatic environment, bioaccumulative, persistent	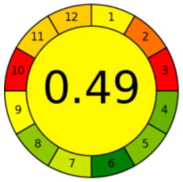
M12	At-line analysis	50 mL	Offline	4 steps (conditioning, extraction, washing, elution)	Semi-automatic (HPLC autosampler)	None	11 mL	No. of analytes:7; throughput:2	LC-UV	None of the reagents from biobased sources	Toxic solvents involved	Toxic to the aquatic environment, bioaccumulative, persistent	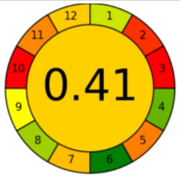

Table S2. Detailed criterion-level assessment of the selected methods using MoGAPI.

No.	Collection	Preservation	Transport	Storage	Type of method	Scale of extraction	Solvents used	Additional treatment	Amount of solvents
M1	Offline	Chemical or physical	Required	Under normal condition	Extraction required	Macro-extraction	Non green solvents	Simple treatment (solvent removal)	<10 mL
M2	Offline	Chemical or physical	Required	Under normal condition	Extraction required	Micro-extraction	Non green solvents	Simple treatment (solvent removal)	<10 mL
M3	Offline	Chemical or physical	Required	Under normal condition	Extraction required	Macro-extraction	Non green solvents	Simple treatment (solvent removal)	10-100 mL
M4	Offline	Chemical or physical	Required	Under normal condition	Extraction required	Micro-extraction	Green solvents/reagents used	Simple treatment (dilution)	<10 mL
M5	Offline	Chemical or physical	Required	Under normal condition	Extraction required	Micro-extraction	Green solvents/reagents used	Simple treatment (dilution)	<10 mL
M6	Offline	Chemical or physical	Required	Under normal condition	Extraction required	Macro-extraction	Non green solvents	Simple treatment (solvent removal)	10-100 mL
M7	Offline	Chemical or physical	Required	Under normal condition	Extraction required	Macro-extraction	Non green solvents	Simple treatment (solvent removal)	<10 mL
M8	Offline	Chemical or physical	Required	Under normal condition	Extraction required	Micro-extraction	Non green solvents	None	<10 mL
M9	Offline	Chemical or physical	Required	Under normal condition	Extraction required	Micro-extraction	Non green solvents	Simple treatment (solvent removal)	<10 mL
M10	Offline	Chemical or physical	Required	Under normal condition	Extraction required	Micro-extraction	Non green solvents	None	<10 mL
M11	Offline	Chemical or physical	Required	Under normal condition	Extraction required	Micro-extraction	Non green solvents	Simple treatment (solvent removal)	<10 mL
M12	On-line or at-line	Chemical or physical	Required	Under normal condition	Extraction required	Macro-extraction	Non green solvents	Simple treatment	10-100 mL

No.	Health hazard	Safety hazard	Energy	Occupational hazard	Waste	Waste treatment	Quantification	Health hazard	Pictogram
M1	Moderately toxic, NFPA=2 or 3	Highest NFPA flammability or instability	>1.5 kWh per sample	Hermetic sealing of analytical process	1-10 mL	No treatment	Yes	Moderately toxic, NFPA=2 or 3	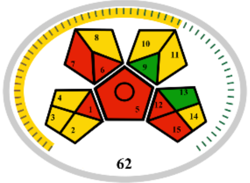
M2	Moderately toxic, NFPA=2 or 3	Highest NFPA flammability or instability	≤0.1 kWh per sample	Hermetic sealing of analytical process	1-10 mL	No treatment	Yes	Moderately toxic, NFPA=2 or 3	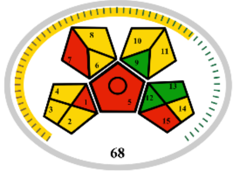

M3	Moderately toxic, NFPA=2 or 3	Highest NFPA flammability or instability	>1.5 kWh per sample	Hermetic sealing of analytical process	>10 mL	No treatment	Yes	Moderately toxic, NFPA=2 or 3	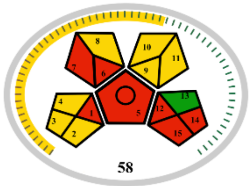
M4	Moderately toxic, NFPA=2 or 3	Highest NFPA flammability or instability	≤0.1 kWh per sample	Hermetic sealing of analytical process	1-10 mL	No treatment	Yes	Moderately toxic, NFPA=2 or 3	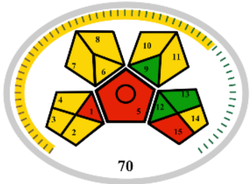
M5	Moderately toxic, NFPA=2 or 3	Highest NFPA flammability or instability	≤0.1 kWh per sample	Hermetic sealing of analytical process	1-10 mL	No treatment	Yes	Moderately toxic, NFPA=2 or 3	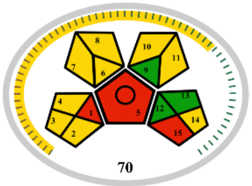
M6	Moderately toxic, NFPA=2 or 3	Highest NFPA flammability or instability	>1.5 kWh per sample	Hermetic sealing of analytical process	>10 mL	No treatment	Yes	Moderately toxic, NFPA=2 or 3	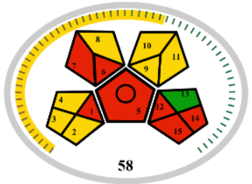
M7	Moderately toxic, NFPA=2 or 3 s	Highest NFPA flammability or instability	≤0.1 kWh per sample	Hermetic sealing of analytical process	1-10 mL	No treatment	Yes	Moderately toxic, NFPA=2 or 3 s	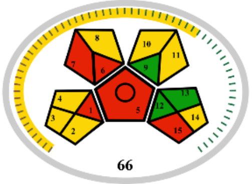
M8	Moderately toxic, NFPA=2 or 3 s	Highest NFPA flammability or instability	>1.5 kWh per sample	Hermetic sealing of analytical process	1-10 mL	No treatment	Yes	Moderately toxic, NFPA=2 or 3 s	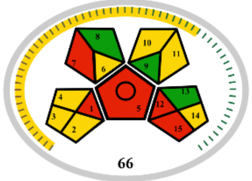

M9	Moderately toxic, NFPA=2 or 3 s	Highest NFPA flammability or instability	≤1.5 kWh per sample	Hermetic sealing of analytical process	1-10 mL	No treatment	Yes	Moderately toxic, NFPA=2 or 3 s	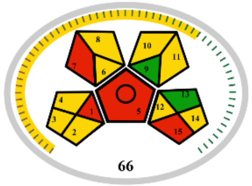
M10	Moderately toxic, NFPA=2 or 3 s	Highest NFPA flammability or instability	>1.5 kWh per sample	Hermetic sealing of analytical process	1-10 mL	No treatment	Yes	Moderately toxic, NFPA=2 or 3 s	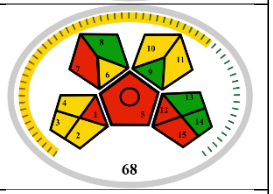
M11	Moderately toxic, NFPA=2 or 3 s	Highest NFPA flammability or instability	≤0.1 kWh per sample	Hermetic sealing of analytical process	1-10 mL	No treatment	Yes	Moderately toxic, NFPA=2 or 3 s	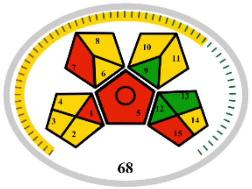
M12	Moderately toxic, NFPA=2 or 3 s	Highest NFPA flammability or instability	≤0.1 kWh per sample	Hermetic sealing of analytical process	>10 mL	No treatment	Yes	Moderately toxic, NFPA=2 or 3 s	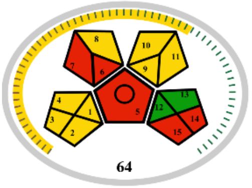

Table S3. Detailed criterion-level assessment of the selected methods using CACI.

#	Criterion 1	Criterion 2	Criterion 3	Criterion 4	Criterion 5	Criterion 6	Criterion 7	Criterion 8	Criterion 9	Criterion 10	Criterion 11	Criterion 12	Criterion 13	Pictogram
	What is the required sample size?	What level of sample preparation is required?	How much time is needed for sample preparation?	Are the chemicals and reagents readily available?	Are the required instruments available in laboratories?	What is the total cost of analysis per sample?:	What is the application type of the method?:	How many analytes can be tested?	How many matrices can be tested?	How portable is the method's instrumentation?	What is the level of automation of this method?	What is the method's sensitivity level?	How long does the sample analysis take?	Score out of 100
M1	>10 mL	Conventional sample preparation	15 min	Commercially available	One special equipment required (SPE manifold and cartridges)	10-100 per sample (SPE cartridges needed)	Quantitative	>3 analytes	2-3 matrices	Not portable nor miniaturized	Semi-automated (LC)	≤10% of the target concentration	15 min	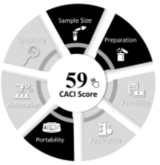
M2	1-10 mL	Conventional sample preparation	20 min	Commercially available	All equipment available in analytical labs	<10$ per sample	Quantitative	>3 analytes	>3 matrices	Not portable nor miniaturized	Semi-automated (LC)	>10% of the target concentration	16 min	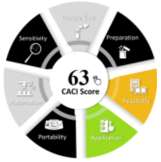
M3	1-10 mL	Conventional sample preparation	3780 min Preparation of organic framework and MSPE	Commercially available	All equipment available in analytical labs	<10$ per sample	Quantitative	>3 analytes	2-3 matrices	Not portable nor miniaturized	Semi-automated (LC)	≤10% of the target concentration	11 min	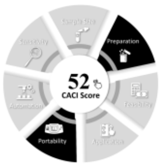
M4	1-10 mL	Conventional sample preparation	12 min	Not commercially available but easily synthesized	All equipment available in analytical labs	<10$ per sample	Quantitative	>3 analytes	2-3 matrices	Not portable nor miniaturized	Semi-automated (LC)	>10% of the target concentration	2 min	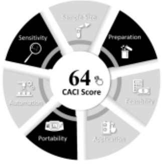
M5	>10 mL	Conventional sample preparation	10.6 min	Not commercially available but easily synthesized	All equipment available in analytical labs	10-100 per sample	Semi-quantitative	>3 analytes	Only one matrix	Miniaturized but not portable	Semi-automated	>10% of the target concentration	15 min	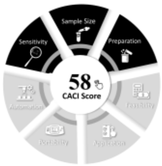
M6	>10 mL	Conventional sample preparation	250 min	Commercially available	One special equipment required (SPE manifold and cartridges)	10-100 per sample (SPE cartridges needed)	Quantitative	>3 analytes	Only one matrix	Not portable nor miniaturized	Semi-automated	≤10% of the target concentration	10 min	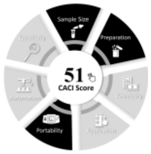

#	Criterion 1	Criterion 2	Criterion 3	Criterion 4	Criterion 5	Criterion 6	Criterion 7	Criterion 8	Criterion 9	Criterion 10	Criterion 11	Criterion 12	Criterion 13	Pictogram
M7	1-10 mL	Conventional sample preparation	132 min	Not commercially available, lab synthesis	All equipment available in analytical labs	10-100 per sample	Quantitative	>3 analytes	Only one matrix	Not portable nor miniaturized	Semi-automated	>10% of the target concentration	5 min	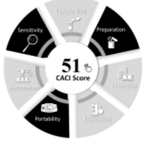
M8	1-10 mL	Conventional sample preparation	2 min	Not commercially available but easily synthesized	One special equipment required (SPE manifold and cartridges	10-100 per sample	Quantitative	>3 analytes	>3 matrices	Not portable nor miniaturized	Semi-automated	≤1% of the target concentration	15 min	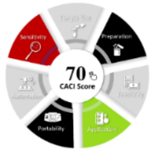
M9	1-10 mL	Conventional sample preparation	5 min	Not commercially available but easily synthesized	One special equipment required (SPE manifold and cartridges	10-100 per sample	Quantitative	>3 analytes	Only one matrix	Not portable nor miniaturized	Semi-automated	>10% of the target concentration	15 min	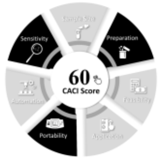
M10	>10 mL	Conventional sample preparation	2220 min	Not commercially available but easily synthesized	More than one special equipment required	10-100 per sample	Quantitative	>3 analytes	>3 matrices	Not portable nor miniaturized	Semi-automated	≤10% of the target concentration	12 min	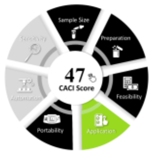
M11	>10 mL	Conventional sample preparation	187 min	Not commercially available but easily synthesized	All equipment available in analytical labs	10-100 per sample	Quantitative	>3 analytes	2-3 matrices	Not portable nor miniaturized	Semi-automated	>10% of the target concentration	30 min	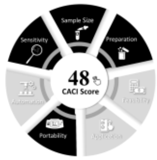
M12	>10 mL	Conventional sample preparation	102 min	Not commercially available but easily synthesized	One special equipment required (3Dprinter)	10-100 per sample	Quantitative	>3 analytes	Only one matrix	Portable	Semi-automated	>10% of the target concentration	30 min	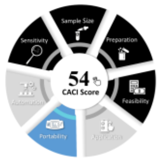

Table S4. Detailed criterion-level assessment of the selected methods using BAGI.

#	Criterion 1	Criterion 2	Criterion 3	Criterion 4	Criterion 5	Criterion 6	Criterion 7	Criterion 8	Criterion 9	Criterion 10	Pictogram
	Type of analysis	Multi- or single-element analysis	Analytical technique	Simultaneous sample preparation	Sample preparation	Samples per h	Reagents and materials	Preconcentration	Degree of automation	Amount of sample	Score out of 100
M1	Quantitative and confirmatory (LC-MS/MS)	Multi-element analysis for 6-15 compounds of the same chemical class (7 analytes)	Instrumentation is not commonly available in most labs (LC-MS/MS)	2-12	Multi-step sample preparation required	5-10	Common commercially available reagents	Preconcentration required after complicated stages	Semi-automated with common devices (e.g. HPLC autosampler)	>100 mL food/environmental (1L)	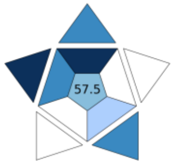
M2	Quantitative	Multi-element analysis for 6-15 compounds of the same chemical class (6 analytes)	Simple instrumentation available in most labs (HPLC-UV)	1	Miniaturized extraction sample preparation	2-4	Common commercially available reagents	Preconcentration required after complicated stages	Semi-automated with common devices (e.g. HPLC autosampler)	<10 mL food/environmental (5 mL)	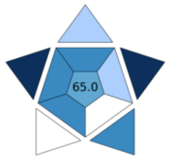
M3	Quantitative	Multi-element analysis for 6-15 compounds of the same chemical class (11 analytes)	Instrumentation is not commonly available in most labs (LC-MS/MS)	1	Miniaturized extraction sample preparation	≤1	Need to be synthesized in the lab with advanced equipment or know how (nanoparticles organic framework)	Preconcentration required after complicated stages	Semi-automated with common devices (e.g. HPLC autosampler)	10.1-50 mL food/environmental (10 mL)	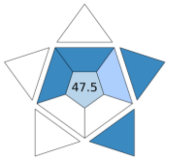
M4	Quantitative	Multi-element analysis for 2-5 compounds of the same chemical class (4 analytes)	Simple instrumentation available in most labs (HPLC-UV)	1	Miniaturized extraction sample preparation	>10	Need to be synthesized in the lab with common instrumentation and in simple way	Preconcentration required after complicated stages	Semi-automated with common devices (e.g. HPLC autosampler)	<10 mL food/environmental (5 mL)	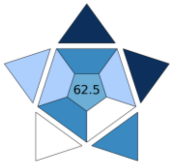
M5	Quantitative	Multi-element analysis for 6-15 compounds of the same chemical class (6 analytes)	Simple instrumentation available in most labs (HPLC-UV)	1	Miniaturized extraction sample preparation	2-4	Common commercially available reagents	Preconcentration required after complicated stages	Semi-automated with common devices (e.g. HPLC autosampler)	10.1-50 mL food/environmental (11 mL)	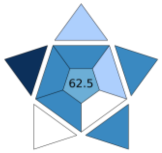
M6	Quantitative and confirmatory (LC-MS/MS)	Multi-element analysis for 6-15 compounds of the same chemical class (7 analytes)	Instrumentation is not commonly available in most labs (LC-MS/MS)	2-12 (SPE)	Multi-step sample preparation required	2-4	Common commercially available reagents	Preconcentration required after complicated stages	Semi-automated with common devices (e.g. HPLC autosampler)	>100 mL food/environmental (1L)	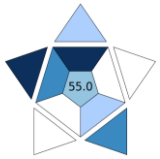

#	Criterion 1	Criterion 2	Criterion 3	Criterion 4	Criterion 5	Criterion 6	Criterion 7	Criterion 8	Criterion 9	Criterion 10	Pictogram
M7	Quantitative	Multi-element analysis for 2-5 compounds of the same chemical class (4 analytes)	Simple instrumentation available in most labs (HPLC-UV)	1	Miniaturized extraction sample preparation	≤1	Need to be synthesized in the lab with advanced equipment or know how (modified nanomaterial)	Preconcentration required after complicated stages	Semi-automated with common devices (e.g. HPLC autosampler)	<10 mL food/environmental (5 mL)	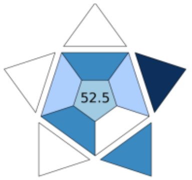
M8	Quantitative and confirmatory (LC-MS/MS)	Multi-element analysis for 2-5 compounds of the same chemical class (4 analytes)	Instrumentation is not commonly available in most labs (LC-MS/MS)	13-95 (SPE)	Miniaturized extraction sample preparation	2-4	Need to be synthesized in the lab with common instrumentation and in simple way	Preconcentration required after complicated stages	Semi-automated with common devices (e.g. HPLC autosampler)	<10 mL food/environmental	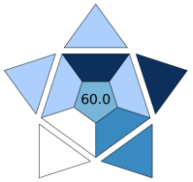
M9	Quantitative	Multi-element analysis for 2-5 compounds of the same chemical class (4 analytes)	Simple instrumentation available in most labs (HPLC-UV)	2-12	Miniaturized extraction sample preparation	2-4	Need to be synthesized in the lab with advanced equipment or know how	Preconcentration required after complicated stages	Semi-automated with common devices (e.g. HPLC autosampler)	10.1-50 mL food/environmental	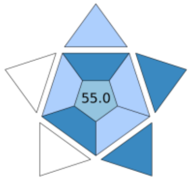
M10	Quantitative and confirmatory (LC-MS/MS)	Multi-element analysis for 6-15 compounds of the same chemical class (10 analytes)	Instrumentation is not commonly available in most labs (LC-MS/MS)	1	Multi-step sample preparation required	1	Need to be synthesized in the lab with advanced equipment or know how	Preconcentration required after complicated stages	Semi-automated with common devices (e.g. HPLC autosampler)	10.1-50 mL food/environmental	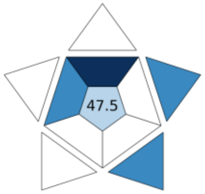
M11	Quantitative	Multi-element analysis for 6-15 compounds of the same chemical class (12 analytes)	Simple instrumentation available in most labs (HPLC-UV)	1	Miniaturized extraction sample preparation	2-4	Need to be synthesized in the lab with advanced equipment or know how	Preconcentration required after complicated stages	Semi-automated with common devices (e.g. HPLC autosampler)	10.1-50 mL food/environmental	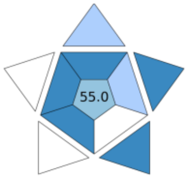
M12	Quantitative	Multi-element analysis for 6-15 compounds of the same chemical class (7 analytes)	Simple instrumentation available in most labs (HPLC-UV)	1	Multi-step sample preparation required	≤1	Need to be synthesized in the lab with advanced equipment or know how	Preconcentration required after complicated stages	Semi-automated with common devices (e.g. HPLC autosampler)	10.1-50 mL food/environmental	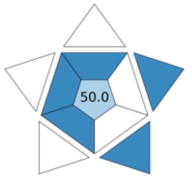

Table S5. Detailed criterion-level assessment of the selected methods using IMOGI.

#	Criterion 1	Criterion 2	Criterion 3	Criterion 4	Criterion 5	Criterion 6	Criterion 7	Criterion 8	Criterion 9	Criterion 10	Pictogram
	To what extent is the analytical process automated, including sample preparation, instrumental analysis, and data processing?	To what extent is the analytical system miniaturized or based on micro-scale or nano-scale technology?	To what extent does the method employ advanced instrumentation for sample preparation or analysis?	To what extent does the method utilize advanced digital tools, data analytics, or artificial intelligence (AI/ML) for analysis, optimization, or interpretation?	How adaptable or scalable is the method for future AI, automation integration, or application to new analytes or matrices	To what extent does the method introduce a novel concept, principle, or mechanism?	To what extent does the method utilize novel reagents, materials, or probes?	To what extent does the method improve efficiency, throughput, or overall performance compared to existing methods?	To what extent does the method integrate knowledge from multiple scientific fields or foster interdisciplinary collaboration?	To what extent does the analytical method consider the principles of green and white analytical chemistry and incorporate recognized evaluation metrics or indexes (e.g. AGREE, AGREEprep, MoGAPI, BAGI, RAPI)?	Score out of 100
M1	Fully automated with minimal human intervention (online SPE)	Conventional large-scale system	Fully advanced technologies utilized (e.g. MS/MS)	Uses computational tools in a limited way	Designed for easy upgrade and integration (LC-MS/MS)	Routine application and applies an established method without significant change	Uses conventional materials or reagents	Substantially faster/more sensitive/higher throughput than conventional methods (high sensitivity)	Stays within a single field with no integration	Does not consider any principles/no metrics are used	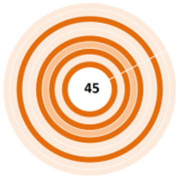
M2	Partially automated	Partially miniaturized	Only conventional technologies (HPLC-UV)	Uses computational tools in a limited way	Adaptable with some modifications	Modifies an existing method in a meaningful way or extends method to new contexts	Adapts known reagents or materials in a slightly innovative way	Comparable or worse performance	Stays within a single field with no integration	Does not consider any principles/no metrics are used	
M3	Partially automated	Partially miniaturized	Fully advanced technologies utilized (e.g. MS/MS)	Uses computational tools in a limited way	Designed for easy upgrade and integration (LC-MS/MS)	Routine application and applies an established method without significant change	Adapts known reagents or materials in a slightly innovative way	Comparable or worse performance	Stays within a single field with no integration	Does not consider any principles/no metrics are used	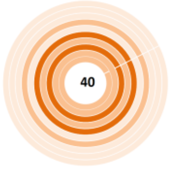
M4	Partially automated	Partially miniaturized	Only conventional technologies (HPLC-UV)	Uses computational tools in a limited way	Adaptable with some modifications	Modifies an existing method in a meaningful way or extends method to new contexts	Uses new reagents/nanomaterials/selective probes (thymol based DES with)	Moderate improvement in method performance (speed/sensitivity/throughput)	Stays within a single field with no integration	Considers all principles/many metrics are used	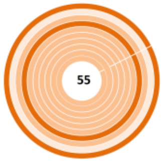
M5	Partially automated	Partially miniaturized	Only conventional technologies (HPLC-UV)	Uses computational tools in a limited way	Adaptable with some modifications (LC-UV)	Modifies an existing method in a meaningful way or extends method to new contexts	Uses new reagents/nanomaterials/selective probes (DES with hydroxyethyl-β-cyclodextrin)	Moderate improvement in method performance (speed/sensitivity/throughput)	Stays within a single field with no integration	Considers few principles/only one or two metrics are used	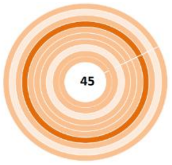

#	Criterion 1	Criterion 2	Criterion 3	Criterion 4	Criterion 5	Criterion 6	Criterion 7	Criterion 8	Criterion 9	Criterion 10	Pictogram
M6	Partially automated	Partially miniaturized	Fully advanced technologies utilized (e.g. MS/MS)	Uses computational tools in a limited way	Designed for easy upgrade and integration (LC-MS/MS)	Routine application and applies an established method without significant change	Uses conventional materials or reagents	Moderate improvement in method performance (speed/sensitivity/throughput)	Stays within a single field with no integration	Does not consider any principles/no metrics are used	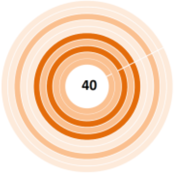
M7	Partially automated	Partially miniaturized	Only conventional technologies (HPLC-UV)	Uses computational tools in a limited way	Adaptable with some modifications (LC-UV)	Modifies an existing method in a meaningful way or extends method to new contexts	Uses new reagents/nanomaterials/selective probes (DES with hydroxyethyl-β-cyclodextrin)	Comparable or worse performance	Stays within a single field with no integration	Considers few principles/only one or two metrics are used	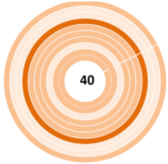
M8	Partially automated	Partially miniaturized	Fully advanced technologies utilized (e.g. MS/MS)	Uses computational tools in a limited way	Designed for easy upgrade and integration (LC-MS/MS)	Routine application and applies an established method without significant change	Adapts known reagents or materials in a slightly innovative way	Substantially faster/more sensitive/higher throughput than conventional methods	Stays within a single field with no integration	Does not consider any principles/no metrics are used	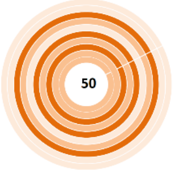
M9	Partially automated	Partially miniaturized	Only conventional technologies (HPLC-UV)	Uses computational tools in a limited way	Adaptable with some modifications (LC-UV)	Routine application and applies an established method without significant change	Adapts known reagents or materials in a slightly innovative way	Comparable or worse performance	Stays within a single field with no integration	Considers few principles/only one or two metrics are used	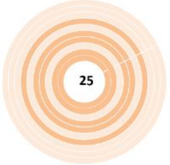
M10	Partially automated	Partially miniaturized	Fully advanced technologies utilized (e.g. MS/MS)	Uses computational tools in a limited way	Designed for easy upgrade and integration (LC-MS/MS)	Routine application and applies an established method without significant change	Uses new reagents/nanomaterials/selective probes (DES with hydroxyethyl-β-cyclodextrin)	Substantially faster/more sensitive/higher throughput than conventional methods	Stays within a single field with no integration	Considers few principles/only one or two metrics are used	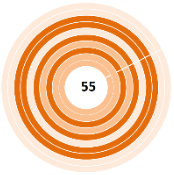
M11	Partially automated	Partially miniaturized	Only conventional technologies (HPLC-UV)	Uses computational tools in a limited way	Adaptable with some modifications (LC-UV)	Routine application and applies an established method without significant change	Adapts known reagents or materials in a slightly innovative way	Moderate improvement in method performance (speed/sensitivity/throughput)	Stays within a single field with no integration	Does not consider any principles/no metrics are used	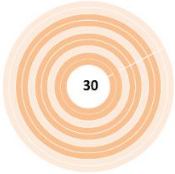
M12	Partially automated	Conventional large-scale system	Only conventional technologies (HPLC-UV)	Uses computational tools in a limited way	Adaptable with some modifications (LC-UV)	Modifies an existing method in a meaningful way or extends method to new contexts	Adapts known reagents or materials in a slightly innovative way	Comparable or worse performance	Stays within a single field with no integration	Does not consider any principles/no metrics are used	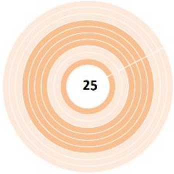

Table S6. Experimental and instrumental characteristics of the selected analytical methods for paraben determination.

No.	Sample volume	Solvent volume	Flow rate	Extraction steps	Chromatographic conditions	Analysis time	Ref.
M1	1 L	8 mL	0.3 mL/min	The filtered water sample was loaded onto the preconditioned cartridge. Then, the SPE cartridges were rinsed and then dried under N2 blowdown. The SPE cartridges were eluted. Then, the collected eluents were concentrated to dryness and reconstituted.	A gradient program: 90% A (0 min), 90% A (1.5 min), 87.5% A (6.5 min), 70% A (9.5 min), 60% A (10.5 min), 10% A (10.7 min), 10% A (11.5 min), 90% A (11.8 min), and finally 90% A (15 min). Injection volume: 5 µL.	15 min	31
M2	5 mL	900 µL of ACN as dispersant and 500 µL of $C_2H_2Cl_4$, 300 µL of 0.4% HAc:ACN	1 mL/min	A mixture containing 900 µL of ACN as dispersant and 500 µL of $C_2H_2Cl_4$ as extractant was injected rapidly into a 10 mL tube containing a 5 mL water sample. The solution was then ultrasonicated for 5 min to form the cloudy solution. Centrifugation was then carried out at 8100 rpm for 8 min. Then, the organic phase deposited at the bottom was transferred into a 5 mL glass vial by using a micropipette. The mixture was dried at 50°C under vacuum and the residue was redissolved into 300 µL of 0.4% HAc:CAN. After filtration, the solution was injected into HPLC.	A gradient elution program started from 16% B, held for 6.8 min, then increased linearly to 60% B within 8 min, and held for 0.2 min before reconditioning. The monitoring wavelength was 270 nm. Injection volume: 10 µL.	14.5 min	32
M3	10 mL	17 mL acetonitrile, for preparation of organic framework: 60 mL ethylene glycol, 80 mL ethanol, 30 mL dioxane, 4.5 mL acetic acids	0.3 mL/min	10 mg of Fe_3O_4@SiO_2@PDE-TAPB-COF was added to 10 mL of the sample solution and vortexed for 20 min. The sorbent was magnetically separated, and the supernatant was discarded. The analytes were desorbed twice with 4.0 mL of acetonitrile each time under vigorous vortexing for 5 min. The combined eluate (8.0 mL) was mixed, and 4.0 mL was evaporated to dryness at 40 °C under N_2. The residue was reconstituted in 1.0 mL of the initial mobile phase, vortexed for 1 min, and filtered through a 0.20 µm membrane before UPLC-MS/MS analysis. The sorbent was regenerated by washing twice with 8.0 mL of acetonitrile under vigorous shaking for 5 min.	The mobile phase is composed of 0.1% formic acid (A) and acetonitrile (B). A linear-gradient elution program was set as follows: (1) 0–3.5 min, 26% B; (2) 3.5– 3.6 min 26–30% B; (3) 3.6–5.8 min, 30% B; (4) 5.8– 5.9 min, 30–90% B; (5) 5.9–7.9 min, 90% B; (6) 7.9– 8.0 min, 90–26% B and (7) 8.0–11.0 min, 26% B.	11 min	33
M4	5 mL	150 µL thymol, 1.5 mL acetonitrile	1 mL/min	An aliquot of 5 mL of each sample (pH adjusted to 4.0) was transferred into a 15-mL polypropylene centrifuge tube, 500 mg NaCl was added, and the solution was vortex mixed. Then, 150 µL of DES was added and the solution was vortex mixed for 2 min. Phase separation was obtained via centrifuging the solution for 10 min at 3500 rpm. The lower aqueous phase was removed using a syringe and the remaining extraction phase was diluted with 150 µL of acetonitrile, vortex mixed, filtered and 2 µL was injected into the UHPLC-DAD.	The mobile phase is composed of 0.1 % formic in water (A) and 90 % acetonitrile in ethanol (B). The gradient elution was as follows: 0–0.5 min: 12 % B, 0.5–1 min: 100 % B, 1–2 min: 12 % B, then hold at 12 % B for 1.5 min for equilibration.	<1.5 min	34
M5	11 mL	81 µL hydroxyethyl-β-	0.8 mL/min	The sample solution was adjusted to pH 4.12 and transferred into a 15-mL plastic conical centrifuge tube.	The mobile phase is composed of 0.1% formic acid (A) and acetonitrile (B); detection	15 min	35

		cyclodextrin, 81 µL methanol		Then, 2.75 g of (NH4)2SO4 was added and the centrifuge tube was gently shaken to dissolve the salt. After the addition of 81 µL hydroxyethyl-β-cyclodextrin DES, the mixture was vortexed for 66 s and centrifuged for 6 min (4,000 rpm, 25 ◦C). After centrifugation, the lower-layer aqueous phase was carefully removed and centrifuged for another 3 min. Then, diluted with equal volume of methanol and filtered prior to injection for HPLC analysis.	wavelength is 285 nm; gradient elution program: 0–8 min, 8–18% B; 8–10.5 min, 18–26% B; 10.5–11.5 min, 26–33% B; 11.5–17.5 min, 33% B; 17.5–25 min, 8% B.		
M6	1 L	6 mL methanol (conditioning), 6 mL 0.1M HCL, 20 mL methanol (elution)	0.3 mL/min	OASIS HLB cartridges were preconditioned with 6 mL methanol, 6 mL distilled water, 6 mL of 0.1 M HCl. Then, 1.0 L of the sample was passed through the cartridge with a flow rate of 4 mL min^{-1}. The cartridge was allowed to dry for 30 min. After drying, the analytes were eluted with 20 mL of methanol. The eluate was evaporated to dryness and re-dissolved in 1 mL of methanol and filtered before injection to LC.	The mobile phase included: (A) 0.1% formic acid in water and (B) acetonitrile. The separation began with 10% solvent B, which increased to 20% for 2 min, and then increased linearly to 60% over 5 min. The conditions as at the beginning of the analysis were reached at 7.1 min. The equilibration time was from 7.1 to 10.0 min.	10 min	36
M7	5 mL	200 µL Methanol for back extraction, 10 mM phosphate buffer and methanol (82:18; v:v)	1 mL/min	5 mL of a water sample was put in a glass vial, and added 200 µL of MNPs@EBH™. The vial was sonicated for 1 min and put to rest on the magnet for 5 min. The supernatant was discarded, and then the solid phase was back-extracted with 200 µL of methanol by sonication for 1 min.	The mobile phase is composed of 10 mM phosphate buffer at pH 7.2 and methanol (82:18; v:v). The injection volume was 20 µL.	5 min	37
M8	10 mL	0.5 mL methanol for activating SPE cartridges and 0.5 mL for elution	0.4 mL/min	The self-made cartridges were installed on a 24-port SPE unit. After being activated with 0.5 mL ultrapure water, 0.5 mL methanol, 10 mL of sample solutions were passed through the adsorbent at a flow rate of 5.0 mL min^{-1}, followed by elution with 0.5 mL elution solvent, and analyzed directly by HPLC-MS/MS	The mobile phase is composed of 0.1% formic acid/water (A) and methanol (B). The elution gradient was as follows: mobile phase B maintained 30% for the first 2.0 min and increased from 30% to 90% linearly for 2.0–6.0 min. From 6.0 to 9.0 min, B remained at 90%. Then, B decreased from 90% to 30% from 9.0 to 9.2 min, and stayed to 15 min.	15 min	38
M9	10 mL	150 µl cetyl trimethyl ammonium chloride, 500 µL methanol, acetonitrile (mobile phase)	Not reported	the procedure of µ-SPE. Briefly, a 5.00 mL empty cartridge prop up by cotton fabric was used to prepare the SPE extraction cartridge by adding 0.4 g silica into the cartridge on cotton fabric. Then, 10 mL of mixed standard/sample solution with 0.03 M CTAB (150 µL) was passed through the SPE cartridge. In this step, sulfonamide-CTAB was adsorbed in silica sorbent. After that, the cartridge was eluted with 500 µL of methanol. Then, 20 µL of eluate was injected into HPLC-PDA system.	Isocratic elution using 25 % acetonitrile in water. The injection volume was 20 µL. The detection of all target analytes was set at 270 nm.	15 min	39
M10	20 mL	0.5 mL methanol	0.25 mL/min	Fabrication of PMA/ MMF-SPME. In the first step, in-situ polymerization in capillaries was adopted to fabricate monolithic fibers. Briefly, 40 mg VBA, 20 mg VPBA, 90	The mobile phase consisted of acetonitrile (ACN, solvent A) and ultrapure water (solvent B). The optimum gradient elution program was as	12 min	40

				mg DVB and 10 mg AIBN were dissolved in 350 mg the mixture of DMSO (245 mg) and 1-propanol (105 mg). The reactants were mixed ultrasonically into a homogenous solution, and high purity nitrogen was employed to remove the oxygen in the solution. Subsequently, the polymerization solution was introduced into a 10 cm length of glass capillary (530 mm in diameter), and then the both ends of the capillary was sealed. The polymerization was performed at 70 C for 12 h. After the reaction, 2.0 cm length of capillary was stripped to get whippy, semitransparent thin PMA fiber. More PMA fibers were prepared according to abovementioned process. In the second step, four PMA fibers were bound at the glass parts to obtain the bunch of PMA/MMF-SPME, and the bunch was dipped in methanol for 24 h to remove the residual monomers.	follows: 0–5.0 min, 10% A; 5.0–5.1 min, 10–30% A, followed by holding at 30% A for 5 min (5.1–10.0 min); 10.0–10.1 min, 30–50% A, followed by holding at 50% A for 5 min (10.1–15.0 min); and 15.0–16.0 min, 50–10% A, followed by holding at 10% A for 12 min (16.0–28.0 min).		
M11	20 mL	2 mL acetonitrile	1 mL/min	20 mL of sample and 3 g of sodium sulfate were stirred at 900 rpm until the salt was completely dissolved and then, 20 mg of zinc ferrites were added, followed by ultrasonication for 15 s. After 30 min of stirring at 900 rpm, the sorbent was washed twice with distilled water. For the desorption of the analytes, 2 mL of acetonitrile, containing 2% v/v formic acid was added after discarding the water and the mixture was ultrasonicated for 30 s. Then, the organic solvent was transferred to an Eppendorf tube and evaporated to dryness, under a gentle nitrogen stream. Finally, 100 µL of water : acetonitrile mixture (90:10 v/v) was added and after ultrasonication for 1 min, a portion of the sample was injected into the HPLC system.	A gradient elution program: water (A) and acetonitrile (B) containing 0.1% (v/v) formic acid. In 30 min, the concentration of B was increased from 10% to 35% and then kept at 35% for 5 more minutes.	30 min	41
M12	50 mL	5 mL acetone, 6 mL methanol	1.2 mL/min	Conditioning step: 3D printed device coated with MCX resin was conditioned with 3 mL methanol and then with 3 mL deionized water for 2 min at 200 rpm. Extraction/preconcentration step: 3D printed device was submerged in the sample or standard solution for 30 min at 200 rpm. Washing step: 3D printed device was rinsed with 5 mL of deionized water. Elution step: 3D printed device was submerged in 3 mL of methanol/ ammonia (95:5, v/v) for 10 min at 200 rpm to the elution of retained SAs.	The mobile phase consisted of 0.1% formic acid in water (A) and acetonitrile (B). Gradient elution conditions started with 95% A and 5% B for 4 min (from 0 to 4 min), followed by a decrease to 60% A and an increase to 40% B for 20 min (from 4.01 to 20 min).	30 min	42

Table S7. Database-specific literature search strategies and study identification.

Database	Web of Science	Scopus	Total
Search platform / collection	Advanced Search; Web of Science Core Collection	Advanced Document Search	—
Search strategy	sulfonamides* environmental water* liquid chromatography	TITLE (sulfonamide*) AND TITLE-ABS-KEY (("environmental water" OR wastewater OR "surface water" OR groundwater OR "drinking water") AND ("liquid chromatography" OR HPLC OR UHPLC OR "LC-MS" OR "LC-MS/MS"))	—
Initial records	300	176	476
Year filter (2020–2026)	115	59	174
Language filter (English)	114	56	170
Final records	114	56	170
